# Agricultural Water Resources Management Using Maximum Entropy and Entropy-Weight-Based TOPSIS Methods

**DOI:** 10.3390/e21040364

**Published:** 2019-04-04

**Authors:** Mo Li, Hao Sun, Vijay P. Singh, Yan Zhou, Mingwei Ma

**Affiliations:** 1 School of Water Conservancy & Civil Engineering, Northeast Agricultural University, Harbin 150030, China; 2 Key Laboratory of Effective Utilization of Agricultural Water Resources of Ministry of Agriculture, Northeast Agricultural University, Harbin 150030, China; 3 Department of Biological and Agricultural Engineering & Zachry Department of Civil Engineering, Texas A & M University, 321 Scoates Hall, 2117 TAMU, College Station, TX 77843-2117, USA; 4 School of Water Conservancy, North China University of Water Resources and Electric Power, Zhengzhou 450046, China

**Keywords:** agricultural water management, supply and demand, optimization and evaluation, maximum entropy, entropy-weight-based TOPSIS

## Abstract

Allocation and management of agricultural water resources is an emerging concern due to diminishing water supplies and increasing water demands. To achieve economic, social, and environmental goals in a specific irrigation district, decisions should be made subject to the changing water supply and water demand—the two critical random parameters in agricultural water resources management. This paper presents the foundations of a systematic framework for agricultural water resources management, including determination of distribution functions, joint probability of water supply and water demand, optimal allocation of agricultural water resources, and evaluation of various schemes according to agricultural water resources carrying capacity. The maximum entropy method is used to estimate parameters of probability distributions of water supply and demand, which is the basic for the other parts of the framework. The entropy-weight-based TOPSIS method is applied to evaluate agricultural water resources allocation schemes, because it avoids the subjectivity of weight determination and reflects the dynamic changing trend of agricultural water resources carrying capacity. A case study using an irrigation district in Northeast China is used to demonstrate the feasibility and applicability of the framework. It is found that the framework works effectively to balance multiple objectives and provides alternative schemes, considering the combinatorial variety of water supply and water demand, which are conducive to agricultural water resources planning.

## 1. Introduction

The conflict between limited water supplies and increased water demands underscores the necessity of efficient and sustainable water resources management. In many counties, irrigated agriculture is the biggest water consumer, occupying more than 70% of available water resources in the world [1,2]. The concept of sustainable agriculture calls for decision-makers to manage water resources not only to focus on economic benefits but also to consider environmental and social effects [3]. Thus, sustainable optimization methods for agricultural irrigation water allocation, which can determine how much water should be allocated to different crops or different regions in obtaining certain goals associated with economic, social, and environmental aspects, is desired and beneficial for agricultural water management.

Water supply and water demand are two main drivers to determine agricultural water resources allocation strategies. In an irrigated agriculture system, water supply is usually derived from upstream runoff (or ground water) and water demand depends highly on crop evapotranspiration. Both runoff and crop evapotranspiration are affected directly by natural conditions and human activities, leading to the randomness of the two parameters [4,5]. Water supply and water demand are closely associated with wet and dry conditions, and their variations have resulted in the sustainable management of agricultural water resources tending to be increasingly vulnerable, particularly in extreme conditions. This emphasizes the importance to investigate the joint probabilities of alternating wet and dry conditions of water supply and water demand, thus guiding the management of agricultural water resources.

Copula functions are useful for deriving joint distributions of two or multiple random variables and are widely used in the field of hydrology and water resources [6,7,8]. For example, Zhang et al. [9] constructed a multivariate copula-based joint probability distribution of water supply and demand with student *t*-copula function based on the data series of precipitation, reference crop evapotranspiration, and irrigation water in the Luhun irrigation district of China. Golian et al. [10] used the copula method to study the joint response of key hydrologic variables, including total precipitation depths and the corresponding simulated peak discharges for different antecedent soil moisture conditions. The formulation of copula function was based on the marginal distributions, and parameter estimation provided a vital role in determining the corresponding functional forms. Many methods have been used to estimate parameters of hydrologic frequency distributions, such as the moments method, maximum likelihood method, probability weighted moments, weight function method, curve-fitting method, maximum entropy principle, Mellin transformation, and minimum interaction entropy method [11,12]. Among them, the maximum entropy principle is widely used for its simple and quick calculation [13].

Further, the sustainable management of agricultural water resources usually involves multiple conflicting objectives, including economic, social, and environmental aspects. That end, multi-objective programming has proven to be an effective way to balance contradictory goals [14,15,16]. How to evaluate the performance of system objectives and the corresponding water resources allocation strategies under different combination scenarios of water supply and demand is conducive to making judicious decisions.

Many methods can be adopted to evaluate system performance. The technique for order preference by similarity to an ideal solution (TOPSIS) is a frequently-used and efficient method for multi-objective decision [17]. TOPSIS is a ranking method approximating the ideal solution and it assesses relative merits for the existing strategies. The determination of weights is needed when using TOPSIS. Entropy-weight-based TOPSIS method is popular, because it only requires the characteristic of monotone increasing/decreasing of different utility functions, and can avoid the subjectivity of weight selection [18]. However, very limited research has been reported on managing agricultural water resources in a comprehensive framework which can achieve trade-off solutions to inform and assist decision-makers by following the steps of uncertainty identification, modeling, optimization, and evaluation.

The objective of this study therefore is to develop a framework that integrates the following components: (1) Determining probability distribution functions of water supply and water demand with parameter estimation using the maximum entropy principle; (2) establishing a joint distribution function of water supply and water demand using a copula function and obtaining their joint occurrence probabilities; (3) modeling agricultural water resources allocation using a multi-objective programming technique; and (4) evaluating system performance under different scenarios based on agricultural water resources carrying capacity using entropy-weight-based TOPSIS method. The framework is then tested in a real case study in an irrigation district in Northeast China.

## 2. Methods

**Figure 1:**
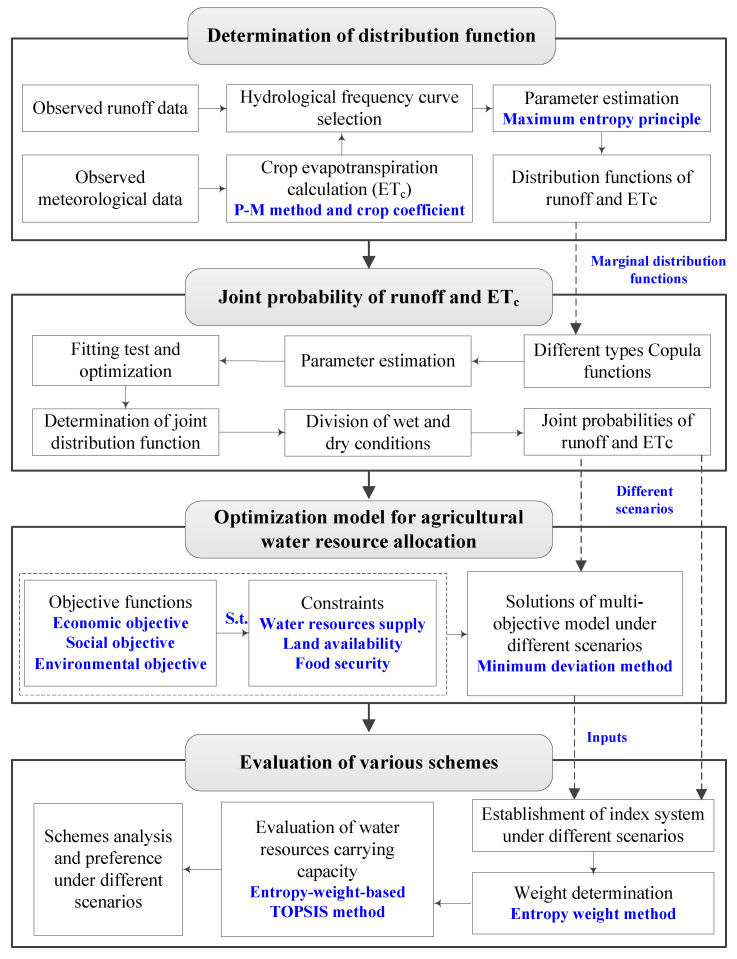
Framework for agricultural water resources management.

This section introduces the methods involving the four components of the proposed framework for agricultural water resources management. Specifically, the aim of the first part is to determine the probability distribution functions of runoff and ET_c_, using the maximum entropy principle to estimate parameters, which is regarded as the marginal distribution function of the joint distribution function of runoff and ET_c_ based on an appropriate copula function (the second part). The output of the second part is the joint occurrence probabilities of runoff and ET_c_. The joint occurrence probabilities are obtained considering the combination and dry, normal, and wet conditions of runoff and ET_c_ based on their joint distribution function, and they are treated as different scenarios for agricultural water allocation and agricultural water resource capacity evaluation. Then, the agricultural water allocation schemes can be obtained under different scenarios (the third part), which will be inputs for the determination of indices for agricultural water resource capacity evaluation. Finally, the evaluation results are obtained under different scenarios (the fourth part). Figure 1 shows the connection of each component and, thereafter, each method involved is described. The dashed line shows the concrete connection of different parts.

### 2.1. Maximum Entropy Principle

Entropy is considered as a measure of information that may be extracted from a system or analogously the uncertainty that the system comprises random events [19], and the uncertainty of a random variable can be described by the probability distribution function. If x denotes a discrete random variable of a system, then the probability when the system in the state $x_{i}(i=1,2,\cdots ,n)$ is expressed as $p\left(x_{i}\right)$. Thus, the entropy of the system with the abbreviation of *H* can be expressed as:

(1) $$H=-\sum_{i=1}^{n}p\left(x_{i}\right)\ln p\left(x_{i}\right)$$

If the random variable is continuous, then *H* can be expressed as:

(2) $$H=-\int_{R}f\left(x\right)\ln f\left(x\right)dx$$

where f(x) is the probability density function of x and *R* is the range of variability of *x*.

Since the maximum entropy principle was introduced in the study of hydrological frequency [20], parameter estimation of hydrological frequency distributions using maximum entropy principle has received much attention. The maximum entropy principle can make the entropy of the known sample data achieve the maximum under given constraints. It can be expressed as:

(3) $$\max H=-\int_{R}f\left(x\right)\ln f\left(x\right)dx$$

(4) $$s.t.\;\int_{R}x^{n}f\left(x\right)dx=\mu_{n},n=1,2\cdots ,N$$

where $\mu_{n}$ is the $n^{th}$ origin moment which is determined using the sample data.

The maximum entropy principle which is used to estimate parameters of hydrological distributions has been found to be accurate. The steps for parameter estimation using the maximum entropy principle can be summarized as:

(1) Calculate the constraints based on the known probability density function, i.e.,:

(5) $$\int_{R}x^{n}f\left(x\right)dx=\mu_{n},n=1,2\cdots ,N$$

(2) Deduce the analytical expression of the probability density function using the maximum entropy distribution that is expressed in terms of Lagrange multipliers. Let $\lambda_{n}\left(n=0,1,\cdots N\right)$ be the Lagrange multipliers. Then the Lagrange function can be expressed as:

(6) $$L=H+\sum_{n=0}^{N}\lambda_{n}\left[\int_{R}x^{n}f\left(x\right)dx-\mu_{n}\right]$$

The variational method was used for deriving f(x). Let the value of δL be equal to 0. Then we have:

(7) $$\begin{array}{l} \delta L=-\int_{R}\left[1+\ln f\left(x\right)\right]\delta f\left(x\right)dx+\sum_{n=0}^{N}\lambda_{n}\int_{R}x^{n}\delta f\left(x\right)dx\\ =\int_{R}\left[-1-\ln f\left(x\right)+\sum_{n=0}^{N}\lambda_{n}x^{n}\right]\delta f\left(x\right)dx \end{array}$$

Due to the arbitrariness of δf(x), the formula in the parentheses of Equation (7) has to be equal to zero. Let $\lambda_{0}$ replace $\lambda_{0}-1$, then we have:

(8) $$\ln f\left(x\right)=\lambda_{0}+\sum_{n=1}^{N}\lambda_{n}x^{n}$$

i.e.,:

(9) $$f\left(x\right)=e^{\lambda_{0}+\sum_{n=1}^{N}\lambda_{n}x^{n}}$$

Equation (9) is the analytical expression of the probability density function based on the maximum entropy.

(3) Deduce the relationship between linear parameters and Lagrange multipliers.

(4) Deduce the relationship between Lagrange multipliers and constraints based on the partial derivative relations between Lagrange multipliers.

(5) Remove Lagrange multipliers and establish the relationship between linear parameters and constraints, i.e., the equation set for parameter estimation, and solve it.

This study conducted the parameter estimation of runoff and crop evapotranspiration (ET_c_) in Jinxi irrigation district in Northeast China. The Pearson III distribution, which is commonly used in China, was selected as the hydrological frequency line. Based on the above steps, the parameter estimation equations for Person III distribution can be expressed as:

(10) E[X]=γ+αβ

(11) $$\sigma^{2}\left(x\right)=\alpha^{2}\beta$$

(12) E[ln(x−γ)]=Ψ(β)+ln(α)

where α, β, and γ are the scale parameter, shape parameter, and location parameter, respectively; E[X] is the mean value; $\sigma^{2}\left(x\right)$ is the variance; and Ψ(β) is the psi function. For parameter estimation equations for other types of hydrological frequency functions, one can refer to [13].

### 2.2. Copula Function

Copula is a multi-dimensional joint distribution function based on marginal distributions and the correlation structure. Assume *X* and *Y* are continuous random variables, with the marginal distributions as $F_{X}$ and $F_{Y}$, respectively. Then, the joint distribution function can be expressed as F(x,y) using a copula function C(u,v) which can be expressed as:

(13) $$C\left(u,v\right)=C^{\theta }\left(F_{X}\left(x\right),F_{Y}\left(y\right)\right)\;\forall x,y$$

where C(u,v) is the copula function and θ is the undermined parameter.

There are many types of copula functions, such as Clayton copula, Gumbel copula, Frank copula, t-copula, Gaussian copula, and Ali-Mikhail-Haq copula [21]. Each type of copula function has its own function structure and parameter estimation method. The merits of different types of copula functions should be judged to select proper functions, and the squared Euclidean distance can be used for the goodness-of-fittest which can be expressed as follows:

(14) $$d^{2}={\sum_{i=1}^{n}\left|{\hat{C}}_{n}\left(u_{i},v_{i}\right)-C\left(u_{i},v_{i}\right)\right|}^{2}$$

Assume $\left(x_{i},y_{i}\right)\left(i=1,2\cdots ,n\right)$ is a sample of the two-dimensional sample of (X,Y), and the empirical distribution functions of X and Y are $F_{n}\left(x\right)$ and $G_{n}\left(y\right)$, respectively. $u_{i}=F_{X}\left(x_{i}\right)$, $v_{i}=F_{Y}\left(y_{i}\right)$, and i=1,2,⋯,n. Then the empirical copula function can be defined as:

(15) $${\hat{C}}_{n}\left(u,v\right)=\frac{1}{n}\sum_{i=1}^{n}I_{\left[F_{n}\left(x_{i}\right)\leq u\right]}I_{\left[G_{n}\left(y_{i}\right)\leq v\right]}$$

where $I_{\left[\;\right]}$ representing the indicative function, and when $F_{n}\left(x_{i}\right)\leq u$, $I_{\left[F_{n}\left(x_{i}\right)\leq u\right]}=1$, otherwise, $I_{\left[F_{n}\left(x_{i}\right)\leq u\right]}=0$. The smaller the value of $d^{2}$, the better the fitting effect.

### 2.3. Optimization Model for Agricultural Water Resources Allocation

This section builds a multi-objective programming model for agricultural water resources allocation, considering economic, social, and environmental aspects. The economic objective is quantified by the system net benefit, the social objective is quantified by the shortage of water resources, and the environmental objective is quantified by the pollutants that fall into the river. In irrigated agricultural systems, water and land can be converted to each other. In order to better reflect the environmental objective, the decision variable of the optimization model was selected as a land resource allocation amount, then the water resources allocation amount can be obtained by multiplying the optimized land resource amount with the corresponding irrigation quota. The objectives of the model can be expressed as follows:

Economic objective

(16) $$f_{Eco}=\max\left(\sum_{i=1}^{n}A_{i}\times Y_{i}\times P-C_{i}\times A_{i}-IQ_{i}\times A_{i}\times C_{a}\right)$$

Social objective

(17) $$f_{Soc}=\min\left(\sum_{i=1}^{n}ET_{ci}\times A_{i}-IQ_{i}\times A_{i}\right)$$

Environmental objective

(18) $$f_{Env}=\min\left(\sum_{i=1}^{n}A_{i}\times \left(\lambda_{COD_{cr}}\times PEI_{COD_{cr}}+\lambda_{NH_{3}-H}\times PEI_{NH_{3}-H}+\lambda_{TN}\times PEI_{TN}+\lambda_{TP}\times PEI_{TP}\right)\right)$$

The above objectives are subjected to the following constraints:

(1) Water availability constraint
  (19) $$\sum_{i=1}^{n}\left(IQ_{i}\times A_{i}\right)\leq Q_{s}\times \eta_{s}+Q_{g}\times \eta_{g}$$
(2) Water demand constraint
  (20) $$\sum_{i=1}^{n}\left(IQ_{i}\times A_{i}\right)\geq WD_{\min}$$
(3) Land availability constraint
  (21) $$A_{i,\min}\leq A_{i}\leq A_{i,\max}\;\forall i$$
(4) Food security constraint
  (22) $$\sum_{i=1}^{n}\left(Y_{i}\times A_{i}\right)\geq \sum_{i=1}^{n}\left(PO_{i}\times Pf\right)$$
  where $f_{Eco}$ (RMB, RMB is the Chinese monetary unit), $f_{Soc}$ (m^3^) and $f_{Env}$ (kg) represent economic objective, and social objective and environmental objective, respectively; *i* is the index of subareas; $A_{i}$ is the irrigation area for subarea *i* and it is the decision variable (ha); $Y_{i}$ is the yield per unit area for subarea *i* (kg/ha); *P* is the market price (RMB/kg); $C_{i}$ is the planting cost (RMB/ha); *IQ_i_* is the irrigation quota for subarea *i* (m^3^/ha); $C_{a}$ is the price of irrigation water (RMB/m^3^); $ET_{ci}$ is the actual evapotranspiration for subarea *i* (m^3^/ha); $\lambda_{CODcr}$, $\lambda_{NH_{3}-H}$, $\lambda_{TN}$, and $\lambda_{TP}$ are the coefficient of chemical oxygen demand, ammonia nitrogen, total nitrogen, and total phosphorus, respectively, that flow into the river; $PEI_{COD_{cr}}$, $PEI_{NH_{3}-H}$, $PEI_{TN}$, $PEI_{TP}$ are the emission per unit area of chemical oxygen demand, ammonia nitrogen, total nitrogen, and total phosphorus, respectively (kg/ha); $Q_{s}$ and $Q_{g}$ are the surface water availability and groundwater availability, respectively; $\eta_{s}$ and $\eta_{g}$ are the utilization efficiency of surface water and groundwater, respectively; $WD_{\min}$ is the minimum water demand (m^3^); *A_i_*_,min_ and $A_{i,\max}$ are the minimum and maximum irrigation areas, respectively, (ha); $PO_{i}$ is the population for subarea *i*; Pf is the food demand per capita (kg/capita).

The above multi-objective programming model can be solved by the minimum deviation method. This method is an improved method of the ideal point method, with the advantage of no consideration of relative importance of various objectives, thus the weight determination can be avoided [22]. Assume a multi-objective programming model is expressed as:

(23) $$F_{m}=\left(f_{m,1}\left(x\right),f_{m,2}\left(x\right),\cdots ,f_{m,n}\left(x\right)\right)$$

where $F_{m}$ contains n objectives which they are expressed as $f_{m,1}\left(x\right),f_{m,2}\left(x\right),\cdots ,f_{m,n}\left(x\right)$, among which, $f_{m,k}\left(x\right)\left(k=1,2,\cdots ,l\right)$ represent the minimum objective functions and $f_{m,k}\left(x\right)\left(k=l+1,l+2,\cdots ,n\right)$ represent the maximum objective functions. Based on the minimum deviation method, the multi-objective programming can be transformed into a single-objective programming model that can be expressed as:

(24) $$\min F_{m}{}^{s}=\sum_{k=1}^{l}\frac{f_{m,k}\left(x\right)-f_{m,k}^{\min}}{f_{m,k}^{\max}-f_{m,k}^{\min}}+\sum_{k=l+1}^{n}\frac{f_{m,k}^{\max}-f_{m,k}\left(x\right)}{f_{m,k}^{\max}-f_{m,k}^{\min}}$$

where $f_{m,k}^{\max}$, $f_{m,k}^{\min}$ are the maximum and minimum values of $f_{m,k}\left(x\right)$. In order to obtain the optimal solution of $F_{m}{}^{s}$, the values of $f_{m,k}^{\max}$ and $f_{m,k}^{\min}$ should not be equal.

### 2.4. Entropy-Weight-Based TOPSIS Method

The entropy-weight-based TOPSIS method was used to evaluate various agricultural water resources allocation schemes under different scenarios by analyzing agricultural water resources carrying capacity. The method avoids the subjectivity of weight determination with no excessive requirement of data sample. This is beneficial to analyze the difference between current situation and perfect state of agricultural water resources carrying capacity, and objectively and thoroughly reflect the dynamic change trend of agricultural water resources carrying capacity [23]. In this study, the relative approach degree that is determined by the entropy-weight-based TOPSIS method was used to evaluate the level of agricultural water resources carrying capacity.

Assume there are *n* evaluation objects, *m* evaluation indices, then the evaluation matrix *X* can be formulated as:

(25) $$X=\left[\begin{array}{cccc} x_{11} & x_{12} & \cdots & x_{1m}\\ x_{21} & x_{22} & \cdots & x_{2m}\\ \vdots & \vdots & \vdots & \vdots \\ x_{n1} & x_{n2} & \cdots & x_{nm} \end{array}\right]$$

where $x_{ij}$ is the index for the $i^{th}$ subarea and $j^{th}$ evaluation index; and *m* and *n* denote the year and the number of evaluation indices, respectively.

In order to make different indices comparable, standardization of each index should be conducted. For positive indices, the equation of standardization can be expressed as:

(26) $$x_{ij}^{\prime }=\frac{x_{ij}-\underset{1\leq j\leq m}{\min x_{ij}}}{\underset{1\leq j\leq m}{\max x_{ij}}-\underset{1\leq j\leq m}{\min x_{ij}}}\;\left(i=1,2\cdots ,n;j=1,2\cdots m\right)$$

For negative indices, the equation of standardization can be expressed as:

(27) $$x_{ij}^{\prime }=\frac{\underset{1\leq j\leq m}{\max x_{ij}}-x_{ij}}{\underset{1\leq j\leq m}{\max x_{ij}}-\underset{1\leq j\leq m}{\min x_{ij}}}\;\left(i=1,2\cdots ,n;j=1,2\cdots m\right)$$

Now the normalized matrix can be expressed as

(28) $$X^{\prime }=\left[\begin{array}{cccc} x_{11}^{\prime } & x_{12}^{\prime } & \cdots & x_{1m}^{\prime }\\ 21_{x}^{\prime } & x_{22}^{\prime } & \cdots & x_{2m}^{\prime }\\ \vdots & \vdots & \vdots & \vdots \\ x_{n1}^{\prime } & x_{n2}^{\prime } & \cdots & x_{nm}^{\prime } \end{array}\right]$$

Then, the weights of each index should be determined and entropy weight method was adopted in this study. It is a comparatively objective method compared with common methods for determining weights, such as analytic hierarchy process and Delphi method. The entropy weight method can determine the weights by calculating the entropy value of indices based on the dispersion degree of data. Thus, first, the information entropy of index, $H_{j}$ <is calculated using the following formula:

(29) $$H_{j}=-k\sum_{i=1}^{m}p_{ij}\ln\left(p_{ij}\right)$$

where k=1/lnm and k>0. ln is the Napierian logarithm. Then, the coefficient of difference $G_{j}$ for the $j^{th}$ evaluation index can be expressed as:

(30) $$G_{j}=1-H_{j}$$

Thus, the weighs *W_j_* can be calculated by:

(31) $$W_{j}=\frac{G_{j}}{\sum_{i=1}^{n}G_{j}}\;\left(j=1,2\cdots ,m\right).$$

Based on the weights, the weight-normalized matrix *T* can be obtained by multiplying $X^{\prime }$ with $W_{j}$ and it is expressed as follows:

(32) $$T=W_{j}\times X^{\prime }=\left[\begin{array}{cccc} w_{1}x_{11}^{\prime } & w_{2}x_{12}^{\prime } & \cdots & w_{m}x_{1m}^{\prime }\\ w_{1}x_{21}^{\prime } & w_{2}x_{22}^{\prime } & \cdots & w_{m}x_{2m}^{\prime }\\ \vdots & \vdots & \vdots & \vdots \\ w_{1}x_{n1}^{\prime } & w_{2}x_{n2}^{\prime } & \cdots & w_{m}x_{nm}^{\prime } \end{array}\right]$$

The positive ideal solution which is constituted by the maximum value of each column of matrix *T*, can be obtained as:

(33) $$R^{+}=\left(R_{1}{}^{+},R_{2}{}^{+},\cdots ,R_{n}{}^{+}\right)=\left(\max T_{i1},\max T_{i2},\cdots ,\max T_{in}\right),\left(i=1,2,\cdots ,n\right)$$

Similarly, the negative ideal solution which is constituted by the minimum value of each column of matrix *T*, can be obtained as:

(34) $$R^{-}=\left(R_{1}{}^{-},R_{2}{}^{-},\cdots ,R_{n}{}^{-}\right)=\left(\max T_{i1},\max T_{i2},\cdots ,\max T_{in}\right),\left(i=1,2,\cdots ,n\right)$$

Then, calculate the Euclidean distances from evaluation object to the positive ideal solution and negative ideal solution using the following equations:

(35) $$D_{i}^{+}=\sqrt{\sum_{j=1}^{m}{\left(T_{ij}-R_{j}^{+}\right)}^{2}}\;\left(i=1,2,\cdots ,n\right)$$

(36) $$D_{i}^{-}=\sqrt{\sum_{j=1}^{m}{\left(T_{ij}-R_{j}^{-}\right)}^{2}}\;\left(i=1,2,\cdots ,n\right)$$

Finally, the relative approach degree of evaluation indices and ideal solutions can be expressed as:

(37) $$R_{i}=\frac{D_{i}^{-}}{D_{i}^{+}+D_{i}^{-}}$$

The agricultural water resources carrying capacity can be evaluated based on the value of $R_{i}$. The larger the value of $R_{i}$, the better the situation of carrying capacity.

## 3. Application

### 3.1. Study Area and Data Acquisition

The developed framework was applied to a real case study in Jinxi irrigation district, Fujin City in Northeast China. The longitude is from 131°30′ to 132°37′, and the latitude is from 46°48′ to 47°14′. The climate of the Jinxi irrigation district belongs to the warm temperate zone with obvious seasonality. Jinxi irrigation district is an important base for food production, and is the key area for high quality rice, soybean, and maize in China. Agriculture is the largest water consumer, accounting for more than 95% of the total water use. Among which, irrigation water use occupies approximately 90%. Both surface water and groundwater are used for water supply to the district for food production. The surface water originates from Songhua River. There are four subareas in Jinxi irrigation district, including Songhuajiang, Jinshan, Huama, and Toulin subareas. The current cultivated land of the irrigation district is about 1.01 × 10^5^ ha. According to the *Project Planning Report of Jinxi Irrigation District*, rice occupies the majority of the cultivated land because of its high quality. Therefore, rice was considered as the study crop, and the aim of the optimization model was to allocate limited surface water and groundwater resources to rice in different subareas.

In order to determine the joint probability of water supply and water demand, data related water supply and demand were required. Surface water supply originated from the runoff of Songhua River, therefore, the runoff data of Songhua River at Jiamusi hydrometric station from 1954 to 2015 were collected. In this study, water demand was the numeric equivalent to the value of crop actual evapotranspiration (ET_c_). The actual evapotranspiration was calculated by multiplying the crop coefficient with reference evapotranspiration (ET_0_). Daily ET_0_ was estimated by the Penmen–Monteith formula [24] based on the meteorological data including wind speed, the highest temperature, the average temperature, the lowest temperature, relative humidity, and sunshine duration from national meteorological network. Monthly value of ET_0_ was obtained by the accumulation of the daily values. The crop coefficient of rice was: 0.38 for May, 0.78 for June, 1.335 for July, 1.06 for August, and 0.45 for September. Social-economic data involved in the optimization model were from the *Feasibility Report of Jinxi Irrigation District*, Yearbook of Fujin City and related references. Specifically, data related to different subareas are listed in Table 1. The emission per unit area of chemical oxygen demand, ammonia nitrogen, total nitrogen, and total phosphorus were 150 kg/ha, 11.85 kg/ha, 171.75 kg/ha, and 65.25 kg/ha, respectively, and the coefficient of chemical oxygen demand, ammonia nitrogen, total nitrogen, and total phosphorus that flow into the river were 0.06, 0.05, 0.04, and 0.01, respectively. The market price of rice was 3.16 RMB/kg, the planting cost of rice was 9589 RMB/ha, the price of irrigation water was 0.15 RMB/m^3^.

### 3.2. Parameter Estimation

**Figure 2:**
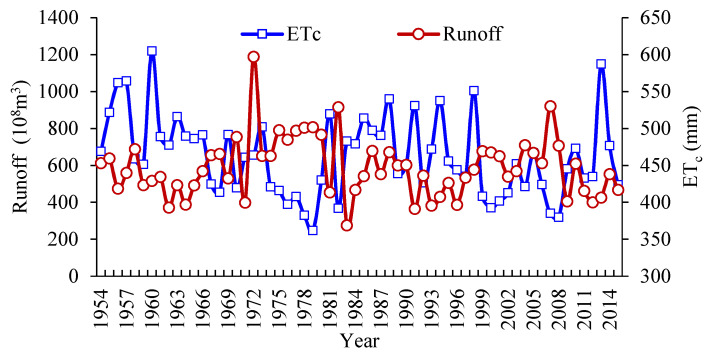
Changes of runoff and ET_c_.

Based on the runoff data of Jiamusi hydrometric station and the method for calculating crop ET_c_, the changes of runoff and ET_c_ from 1954 to 2015 are depicted in Figure 2. In this study, the Pearson III distribution function was used to describe the hydrological distribution of runoff and ET_c_. Based on the maximum entropy principle for Pearson III distribution, the program was coded in MATLAB (MathWork, Natick, MA, USA) and the related parameters were estimated. The scale parameter, shape parameter and location parameter for annual runoff were 12.08, 3.47, and 14.97, respectively. Thus, the distribution of runoff was expressed as:

(38) $$F\left(x\right)=\frac{1}{12.08\Gamma \left(3.47\right)}\int_{0}^{\infty }{\left(\frac{x-14.97}{12.08}\right)}^{2.47}e^{-}{}^{\left(\frac{x-14.97}{12.08}\right)}dx$$

The scale parameter, shape parameter and location parameter for ET_c_ were 15.27, 6.94, and 342.66, respectively. Thus, the distribution of ET_c_ was expressed as:

(39) $$F\left(x\right)=\frac{1}{15.27\Gamma \left(6.94\right)}\int_{0}^{\infty }{\left(\frac{x-342.66}{15.27}\right)}^{5.94}e^{-}{}^{\left(\frac{x-342.66}{15.27}\right)}dx$$

These two distributions were considered as the marginal distributions of the joint distribution function of runoff and ET_c_.

### 3.3. Joint Probability of Water Supply and Water Demand

From Figure 2, the variability between runoff and ET_c_ was obvious, leading to the necessity to obtain the joint probability of wet and dry conditions of these two random variables, thus guiding agricultural water resources allocation. Assume both runoff and ET_c_ had three hydrological characteristics: wet, normal and dry conditions. In this study, the cumulative probability of wet and dry conditions were pw=25%, pd=75%. Specifically, for the wet condition: $X\geq x_{w}$, for the normal condition: $x_{d}\leq X\leq x_{w}$, and for the dry condition: $X\leq x_{d}$, where *X* was the runoff volume or the value of ET_c_, and $x_{w}$ and $x_{d}$ were the critical values between wet and normal conditions, and between normal and dry conditions, respectively. Based on the above principle, there were in total nine scenario combination of runoff and ET_c_, i.e., wet (runoff) vs. wet (ET_c_), wet (runoff) vs. normal (ET_c_), wet (runoff) vs. dry (ET_c_), normal (runoff) vs. wet (ET_c_), normal (runoff) vs. normal (ET_c_), normal (runoff) vs. dry (ET_c_), dry (runoff) vs. wet (ET_c_), dry (runoff) vs. normal (ET_c_), and dry (runoff) vs. dry (ET_c_). Based on the marginal distribution functions of runoff and ET_c_, this study selected the commonly used Gaussian copula, t-copula, Archimedean copula (including Clayton copula, Frank copula, Gumbel copula, and Ali–Mikhail–Haq copula in this study which are frequently-used in hydrology-related analysis) to establish the joint distribution functions. The specific equations of these copula functions are presented in Appendix A. In this study, the Euclidean distance ($d^{2}$), which is a non-probabilistic measure of goodness of approximation with the advantage of simple calculation [25,26], was used to evaluate the performance of each selected copula function. The value of $d^{2}$ was 0.042, 0.0411, 0.2037, 0.0402, 0.2037, and 0.3232 corresponding to the above copula functions. Results showed that the value of $d^{2}$ of Frank copula was the smallest, thus, the Frank copula was adopted and the joint distribution function of runoff and ET_c_ could be expressed as [27]:

(40) $$C\left(U,V\right)=-\frac{1}{\theta }\ln\left[1+\frac{\left(e^{-\theta U}-1\right)\left(e^{-\theta V}-1\right)}{\left(e^{-\theta }-1\right)}\right]$$

where *U* indicated the runoff, *V* indicated the ET_c_. In this study, θ=−3.595.

**Figure 3:**
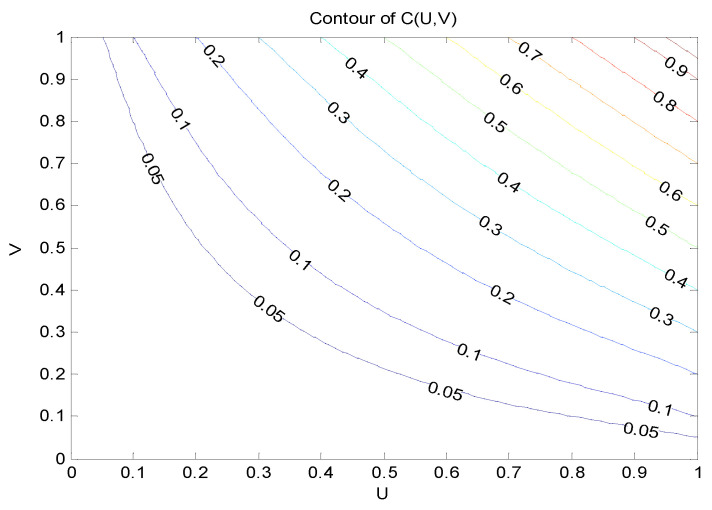
Joint probability of runoff (*U*) and ET_c_ (*V*).

Based on the joint distribution function, the contour of C(u,v) were drawn as Figure 3 shows, and the joint probabilities of each scenario was obtained. For example, the joint probability for the scenario of wet (runoff) vs. wet (ET_c_) was 1.6%, for the scenario of dry (runoff) vs. dry (ET_c_) was 1.63%, and for the scenario of normal (runoff) vs. normal (ET_c_) was 28.15%. The synchronous joint probability of runoff and ET_c_ (31.38%) was appreciably lower than the asynchronous joint probability of runoff and ET_c_ (68.62%). The runoff values corresponding to wet, normal, and dry conditions were 754.845 × 10^8^ m^3^, 604.136 × 10^8^ m^3^, and 474.903 × 10^8^ m^3^, respectively. The ET_c_ values corresponding to wet, normal and dry conditions were 472.27 mm, 443.54 mm and 419.41 mm, respectively. The proportion of water supply for Jinxi irrigation district of the runoff volume from Jiamusi hydrometric station were 0.66% for wet condition, 0.7% for normal condition and 0.74% for dry condition. Therefore, the values of water supply and water demand under each scenario were obtained.

### 3.4. Agricultural Water Resource Allocation Schemes

**Figure 4:**
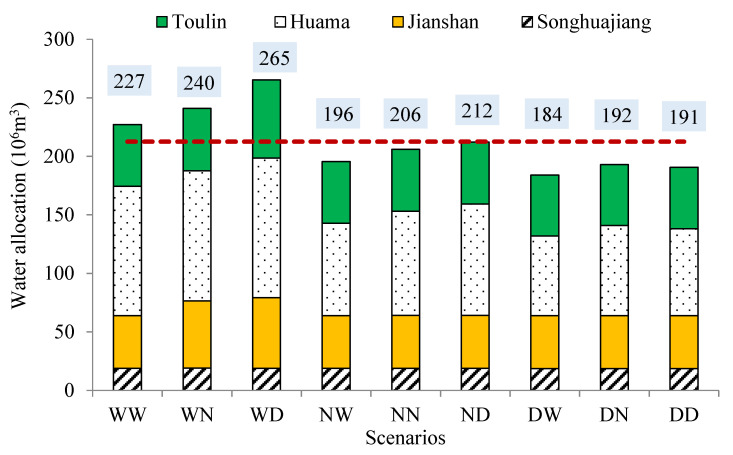
Water allocation for different subareas under different scenarios. (Note: (1) W—wet, N—normal, D—dry; (2) the first letter indicates the conditions of runoff and the second letter indicates the conditions of ET_c_; (3) the number inside the box means the total water allocation amount of different scenarios; and (4) the red dashed line is the average value of different scenarios).

According to the minimum deviation method, the optimization model was solved. The irrigation area was allocated to different subareas, then the water allocation amount can be obtained, based on the irrigation quota of different subareas, as shown in Figure 4. From the figure, it was obvious that if the wet and dry conditions of runoff were considered only, the water allocation amounts followed the law: wet > normal > dry conditions, indicating that a larger water supply would lead to a larger water allocation. If the wet and dry conditions of water demand were considered only, the water allocation amounts followed the law: dry > normal > wet conditions, indicating that a larger water demand would lead to a larger water allocation, because the value of water demand in the wet condition was the smallest, while it was the highestin the dry condition. Hence, two extreme conditions happened in the WD and DW scenarios between which DW indicated the most water shortage condition and should draw attention of policymakers, with the corresponding water allocation amount being 1.96 × 10^8^ m^3^. Based on the joint probabilities of different scenarios, the average water allocation level was obtained, i.e., 2.13 × 10^8^ m^3^. Such a result could provide a guidance in water resource planning of Jinxi irrigation district considering the combination of different scenarios. For different subareas, the change in the water allocation amount in Huama subarea was obvious because of its larger irrigation area. Based on the optimal results, the value of economic objective ranged from 8.32 × 10^8^ RMB to 12 × 10^8^ RMB, with the average value under the nine scenarios being 9.55 × 10^8^ RMB. The value of social objective ranged from 3.29 × 10^7^ m^3^ to 7.13 × 10^7^ m^3^, with the average value under the nine scenarios being 5.05 × 10^7^ m^3^. The value of environmental objective ranged from 1.67 × 10^6^ kg to 2.39 × 10^6^ kg, with the average value under the nine scenarios being 1.91 × 10^6^ kg. These were the results of a comprehensive coordination of the three objectives.

### 3.5. Agricultural Water Resources Carrying Capacity

**Figure 5:**
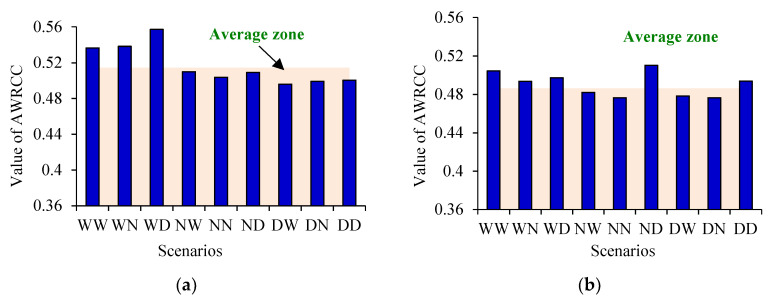

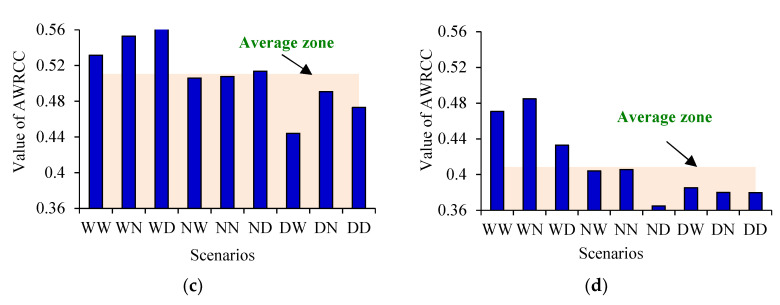
Agricultural water resource carrying capacity for different subareas. (**a**) Songhuajiang; (**b**) Jinshan; (**c**) Huama; and (**d**) Toulin. (Note: AWRCC means agricultural water resource carrying capacity).

Based on the results of optimization model, the agricultural water resource carrying capacity was evaluated using the entropy-weight-based TOPSIS method. As the optimization model considered three objectives, involving economic, social, and environmental factors, the index system for evaluating agricultural water resources carrying capacity contained three dimensions, i.e., economic, social, and environmental dimensions, and each dimension contained three parameters. Detailed information of the index system can be found in Table 2. Acquisition of the values of these indices was based on the results of the optimization model and actual conditions. Using the entropy-weight method, the weight of each index was calculated as shown in Table 1. Based on the entropy-weight-based TOPSIS method, the agricultural water resources carrying capacity values for the four subareas were obtained as shown in Figure 5 This study divided the value of agricultural water resources carrying capacity into five grades: [0, 0.2) belonged to I grade, [0.2, 0.4) belonged to II grade, [0.4, 0.6) belonged to III grade, [0.6, 0.8) belonged to IV grade, and [0.8, 1.0) belonged to V grade. The larger the value the higher the grade, indicating the better the agricultural water resources carrying capacity. From Figure 5, the agricultural water resources carrying capacity in Toulin subarea was the worst with the average value of the nine scenarios being 0.41, and in Songhuajiang subarea it was the best with the average value of the nine scenarios being 0.52. However, the fluctuations of agricultural water resources carrying capacity for Huama subarea and Toulin subarea were obvious under different scenarios. This indicated that the agricultural water resources carrying capacity in these two subareas was sensitive to the changes of water supply and water demand. In general, the resources carrying capacity in the wet condition in terms of runoff was higher than that in the dry condition, indicating that the resources carrying capacity was more sensitive to water supply. Therefore, improving water use efficiency under the limited water supply was significant.

### 3.6. Discussion

Compared with traditional irrigation water allocation modes which were obtained based on “Water Resources Verification Report of Jinxi Irrigation District”, “Water Resources Argumentation Report of Jinxi Irrigation District”, and “Engineering Feasibility Study Report of Jinxi Irrigation District”, one of the main contributions of this study was to consider the changes of water supply and water demand as well as their interrelationships. For this, we established the joint distribution function of water supply and water demand, based on which, different scenarios with the combination of wet and dry conditions of water supply and water demand were generated. This would provide decision makers more reasonable references for irrigation water allocation. Additionally, this could help decision-makers for irrigation water allocation to response promptly to different changes in natural conditions, andimprove the adaption ability to climate change. Another major contribution of this study was that multiple targets were considered when optimally allocating limited water resources, including economic, social and environmental aspects. This avoided the disadvantage of traditional irrigation water allocation patterns of the study area that focused on economic benefit. (In the related reports as mentioned above, the current irrigation water allocation plans for crops were based on the benefit-cost analysis, without analyzing the associated social and environmental impacts). Compared with actual conditions of the study area, taking the normal-normal scenario of water supply and water demand which was the scenario that was the most likely to happen (with the joint probability was 28.15%) as an example, the optimal results could save 0.57 × 10^8^ m^3^ water resources. From the angle of environmental protection, the optimal results could reduce pollutants emission of 5.14 × 10^5^ kg. Such results were conducive to improve the efficiency and sustainability of irrigation water allocation. Moreover, the agricultural water resource carrying capacity was evaluated based on the optimal results to help decision makers evaluate the irrigation strategies under different scenarios and, thus, provide guidance when planning the irrigation water of the irrigation district. The major limitation of this study was that the dynamics of the framework was ignored, for example, the changes of water supply and demand during the crop growth period and the changes in crop growth factors were not considered, which might affect the applicability of the proposed framework. Future work would make it a priority to improve the proposed framework.

## 4. Conclusions

This study developed a framework for agricultural water resources management, including the determination of the distribution function of water supply and water demand based on parameter estimation, joint probability of water supply and water demand, optimization model for agricultural water resources allocation, and evaluation of agricultural water resources carrying capacity. The relationship between the adjacent components is input-output. Specifically, because of the characteristic of high accuracy and quick calculation, the maximum entropy methodis used to estimate the parameters of the probability distributions of water supply and water demand, so that the joint probabilities of these two random variables can be obtained based on the copula function. Thus, nine scenarios with the combination of wet and dry conditions of water supply and water demand are generations, and agricultural water resources is optimally allocated under these scenarios, considering the comprehensive benefit of economic, social, and environmental dimensions. The results can provide decision makers more alternatives of water allocation schemes considering the changes of natural conditions, which is conducive to agricultural water planning. Lastly, agricultural water resources carrying capacity is evaluated based on the optimal results using entropy-weight-based TOPSIS methods, which can avoid the subjectivity of weight determination, and objectively and thoroughly reflect the trend of dynamic change of agricultural water resources carrying capacity.

The developed framework is applied to a real case study in an irrigation district in northeast China. Results demonstrated the feasibility and applicability of using the maximum entropy method and entropy-weight-based TOPSIS method to agricultural water resources management, and the results can provide a certain guidance for local agricultural water management. The developed framework and the associated method can also be applied to regional resources management problem or to other regions. This study aims to construct such a framework for agricultural water resources management, however, concrete details for some components are simplified, for example, the objective functions and constraints of optimization model are simple with only basic elements associated with economic, social, and environmental aspects being considered, and the dynamics of the framework are overlooked. These deserves further study to improve the framework.

## Figures and Tables

**Table 1:** Data related to different subareas.

Parameter	Unit	Subarea			
		Songhuajiang	Jinshan	Huama	Toulin
Yield per unit area	kg/ha	8465.67	8511.17	8511.17	7887.33
Irrigation quota	m^3^/ha	3660.33	3686.48	3686.48	3327.95
Population	10^4^ people	0.77	4.31	1.12	1.62
Maximum irrigation area	10^4^ ha	0.53	1.85	3.34	2.28
Minimum irrigation area	10^4^ ha	0.51	1.225	1.85	1.43

**Table 2:** Index system and weights.

Dimension	Index	Calculation Formula	Unit	Index Attribute	Weights
Economic dimension (A)	Water production efficiency (A1)	Yield/(Crop evapotranspiration)	kg/ha	+	0.1013
	Production value per unit water (A2)	(Yield per unit water) × Market price	RMB/m^3^	+	0.1095
	Grain output (A3)	Yield × (Market price)	RMB	+	0.1018
Social dimension (B)	Food per capita (B1)	Yield/Population	kg/capita	+	0.1443
	Water per capita (B2)	Water resource amount/Population	m^3^/capita	+	0.0923
	Agricultural water shortage (B3)	(Crop evapotranspiration-Irrigation amount) × Irrigation area	m^3^	−	0.1393
Environmental dimension (C)	Agricultural non-point pollution discharge (C1)	(Emission of agricultural non-point pollution per unit area) × Planting area	kg	−	0.0996
	Agricultural greenhouse gases emission (C2)	(Emission of agricultural greenhouse gases per unit area) × Planting area	kg	−	0.0997
	Coefficient of groundwater exploitation (C3)	(Groundwater exploitation amount)/(Total groundwater amount)	%	−	0.1122

Note: “+” indicates the index belongs to the attribute of “the larger, the better”, while “−” indicates the index belongs to the attribute of “the smaller, the better”.

## Appendix A

**Table A1:** Equations of the copula functions in this study.

Function Name	C(u,v)	Interpretation
Gaussian copula	$\int_{-\infty }^{\phi^{-1}\left(u\right)}\int_{-\infty }^{\phi^{-1}\left(v\right)}\frac{1}{2\pi \sqrt{1-\rho^{2}}}\exp\left[-\frac{s^{2}-2\rho st+t^{2}}{2\left(1-p^{2}\right)}\right]dsdt$	$\phi^{-1}$ is the inverse function of standard normal distribution function; ρ is the correlation coefficients between variables.
t-copula	$\int_{-\infty }^{t_{k}{}^{-1}\left(u\right)}\int_{-\infty }^{t_{k}{}^{-1}\left(v\right)}\frac{1}{2\pi \sqrt{1-\rho^{2}}}\left[1+\frac{s^{2}-2\rho st+t^{2}}{k\left(1-p^{2}\right)}\right]dsdt$	$t_{k}^{-1}$ is the inverse function t distribution function with the degree of freedom is k; ρ is the correlation coefficients between variables.
Clayton copula	${\left(u^{-\theta }+v^{-\theta }-1\right)}^{-1/\theta }$	θ>0 and $\tau =\frac{\theta }{\theta +2}$. τ is the Kendall coefficient of rank correlation, and the same below.
Frank copula	$-\frac{1}{\theta }1n\left[1+\frac{\left(e^{-\theta u}-1\right)\left(e^{-\theta v}-1\right)}{e^{-\theta }-1}\right]$	θ∈R and $\tau =1-\frac{4}{\theta }\left[-\frac{1}{\theta }\int_{\theta }^{0}\frac{t}{\exp\left(t\right)-1}dt-1\right]$
Gumbel copula	$\exp\left[-{\left({\left(-1nu\right)}^{\theta }+{\left(-1nv\right)}^{\theta }\right)}^{1/\theta }\right]$	θ≥1 and $\tau =1-\frac{4}{\theta }\left[-\frac{1}{\theta }\int_{\theta }^{0}\frac{t}{\exp\left(t\right)-1}dt-1\right]$
Ali–Mikhail–Haq copula	$\frac{uv}{1-\theta \left(1-u\right)\left(1-v\right)}$	θ∈[−1,1) and $\tau =\left(\frac{3\theta -2}{\theta }\right)-\frac{2}{3}{\left(1-\frac{1}{\theta }\right)}^{2}1n\left(1-\theta \right)$
